# Regulation and Subfunctionalization of Flowering Time Genes in the Allotetraploid Oil Crop *Brassica napus*

**DOI:** 10.3389/fpls.2020.605155

**Published:** 2020-11-20

**Authors:** Sarah Schiessl

**Affiliations:** ^1^Department of Plant Breeding, IFZ Research Centre for Biosystems, Land Use and Nutrition, Justus Liebig University Giessen, Giessen, Germany; ^2^Department of Botany and Molecular Evolution, Senckenberg Research Institute and Natural History Museum Frankfurt, Frankfurt, Germany

**Keywords:** polyploidy, gene evolution, subfunctionalization, flowering time, canola, oilseed rape

## Abstract

Flowering is a vulnerable, but crucial phase in building crop yield. Proper timing of this period is therefore decisive in obtaining optimal yields. However, genetic regulation of flowering integrates many different environmental signals and is therefore extremely complex. This complexity increases in polyploid crops which carry two or more chromosome sets, like wheat, potato or rapeseed. Here, I summarize the current state of knowledge about flowering time gene copies in rapeseed (*Brassica napus*), an important oil crop with a complex polyploid history and a close relationship to *Arabidopsis thaliana*. The current data show a high demand for more targeted studies on flowering time genes in crops rather than in models, allowing better breeding designs and a deeper understanding of evolutionary principles. Over evolutionary time, some copies of rapeseed flowering time genes changed or lost their original role, resulting in subfunctionalization of the respective homologs. For useful applications in breeding, such patterns of subfunctionalization need to be identified and better understood.

## Introduction

### Rapeseed for Future

In a future fossil-free mobility strategy, plant-based fuels cannot fully replace fossil fuels, as the production quantity of plant oils is by far too low (Carlsson et al., 2011), but they may be used in all cases where electricity-based machines cannot provide sufficient power, like tractors or harvesting machines (Bender, 1999). Plant-based fuels will therefore become an important building block in decarbonizing agriculture. The most popular plant-based fuel in temperate areas is biodiesel, which is mostly produced from rapeseed oil (Bušić et al., 2018). Although rapeseed oil is also a healthy edible oil, its use for fuel is dominating. Rapeseed is grown all across the globe in different climate zones from boreal to subtropical climates and constitutes the second most important oil crop of the world after soybean, comparable to oil palm (FAOSTAT, sourced July 2020). Breeding has formed three distinct oilseed rape growth types: spring rapeseed, which is grown as an annual, mostly in Australia and Canada, semi-winter rapeseed, which gives better yield but has an increased vegetation period, and winter rapeseed, which is biennial and yields best of all three types. Growth type is largely determined by flowering behavior and winter hardiness. Winter types are winter-hardy and depend on a period of prolonged cold to attain the ability to flower (vernalization), while semi-winter types are less vernalization dependent and lack winter hardiness, and spring types lack both traits (Schiessl et al., 2017b). Besides those oilseed forms, a further subspecies, ssp. *napobrassica*, is cultivated as beets and known as swedes or rutabagas. Swedes are generally strongly vernalization-dependent, but lack strong winter hardiness. Due to the importance of those traits, each growth type usually constitutes its own breeding pool. However, breeding for other traits, mainly oil and seed quality, has strongly reduced genetic diversity within the breeding pools, and cross-breeding between pools might be one solution to increase genetic diversity and increase breeding gains (Snowdon and Iniguez Luy, 2012). Shifts in climate zones due to climate change may also demand growth type adaptation in future (IPCC, 2019).

### Why Timing of Flowering Does Matter

In most plants and also in oilseed rape, flowering is the most sensitive phase for yield building due to various reasons: the increased energy demand due to flower formation (Borghi et al., 2019), but also due to shadowing of the leaves by the flowers, reducing photosynthesis (Diepenbrock, 2000). Flowering time is also critical in terms of nitrogen use efficiency, as N uptake after flowering is strongly correlated to yield (Berry et al., 2010). Moreover, drought stress during flowering was found to be much more devastating than during vegetative development (Hohmann, 2017). Many rapeseed growing areas face increased likelihoods of droughts during rapeseed flowering times due to climate change (Lu et al., 2019). If the drought period is short, drought avoidance including later flowering can be a successful strategy (Schiessl et al., 2020), but this is highly dependent on synchronization of drought period and flowering time. In winter oilseed rape, flowering time is also a general obstacle to breeding progress. Winter oilseed rape requires long periods of 6 to 10 weeks of vernalization to achieve flowering competence, leading to long generation times. In other crops and in spring rapeseed, attempts to shorten generation times in growth chambers were successful using special light regimes (Ghosh et al., 2018; Watson et al., 2018); however, this remains to be achieved for winter rapeseed. Finally, oilseed rape is a facultative outcrossing species and therefore not strictly dependent on pollinators, however, the presence of pollinators in the field normally increase seed yield and quality (Bommarco et al., 2012; Zou et al., 2017; Adamidis et al., 2019). It is thus helpful to synchronize flowering time with pollinators’ activity. Flowering time has therefore an important influence on many agronomic traits and environment-specific flowering time adaptation needs to be carried out in individual breeding programs to maximize yield.

## What Is Different to *Arabidopsis thaliana*?

*Brassica napus* is a close relative to the model crucifer *Arabidopsis thaliana*. Most *A. thaliana* flowering time genes are conserved in the species (Osborn et al., 1997), indicating that flowering time regulation in oilseed rape is regulated in a similar way as in the model system. There are, however, species-specific drawbacks in this conclusion which are mainly due to the polyploid nature of the *B. napus* genome. *B. napus* is a recent allotetraploid carrying a subgenome A from the donor species *B. rapa* as well as a subgenome C from the donor species *B. oleracea* (Morinaga, 1934; Nagaharu, 1935). *B. rapa* and *B. oleracea* are two closely related species separated by around 4.5 Mio years of evolution, going back to a common hexaploid ancestor (Schiessl and Mason, 2020). This evolutionary history raises the theoretical number of homologs to six, although gene loss has reduced the average copy number to 4.4 (Parkin et al., 2010). For the main flowering time regulators, the average copy number was found to be 4.8, ranging from one to up to twelve copies (Schiessl et al., 2014). Moreover, the individual copy number in flowering time was found to be highly variable in a representative diversity set, although this did not affect all copies equally (Schiessl et al., 2017b). At the same time, several transcriptomic studies have shown that expression patterns vary a lot between copies of the same *Arabidopsis* homolog, with different expression maxima, different tissue or age specificity or different reactivity to stress (Zhou et al., 2007; Guo et al., 2014; Shah et al., 2018; Kittipol et al., 2019; Schiessl et al., 2019a). Together, this indicates that flowering time genes in *B. napus* underwent considerable subfunctionalization. To identify useful breeding targets, it is therefore crucial to identify the conserved or acquired role of each flowering time gene copy individually. In the next paragraphs, I will summarize the progress of such attempts for the different regulatory modules identified in *Arabidopsis*. A list of *B. napus* homologs detected within QTL, GWAS peaks or located in selective sweeps is given in Table 1. For general reviews about flowering time regulation in models and crops, please refer to the excellent works of others in this area (Jung and Muller, 2009; Srikanth and Schmid, 2011; Blümel et al., 2015).

**TABLE 1:** List of most important flowering time genes with respective QTL, GWAS peaks or within selective sweeps in *B. napus* with chromosomal locations and references.

**Gene name in AT**	**Abbreviated as**	**Candidate in QTL study**	**Candidate in GWAS**	**Candidate in selective sweep analysis**	**References**
*FLOWERING LOCUS T*	*FT*	A02, A07ab	A02	A02	Raman et al., 2013, 2019; Nelson et al., 2014; Wu et al., 2018
*FLOWERING LOCUS D*	*FD*			C01, C03	Schiessl et al., 2017b
*FLOWERING LOCUS C*	*FLC*	A02, A03ab, A10, C02, C03	A02, A10, C03	A10, C03	Quijada et al., 2006; Udall et al., 2006; Fletcher et al., 2014; Nelson et al., 2014; Raman et al., 2016; Schiessl et al., 2017b; Wu et al., 2018
*FRIGIDA*	*FRI*	A03	A03	A03	Wang et al., 2011; Raman et al., 2013, 2016; Schiessl et al., 2015, 2017b
*VERNALIZATION INSENSITIVE 3*	*VIN3*	A02, A03		A02	Shi et al., 2009; Nelson et al., 2014; Schiessl et al., 2017b; Shah et al., 2018
*FY*	*FY*	C02			Jian et al., 2019
*CONSTANS (-like)*	*CO (-like)*	A02, A10, C01, C03, C09	A02, A03, C09	A10, C09	Quijada et al., 2006; Nelson et al., 2014; Xu et al., 2016; Schiessl et al., 2017b; Li et al., 2018; Rahman et al., 2018
*SENSITIVITY TO RED LIGHT REDUCED 1*	*SRR1*			A02	Schiessl et al., 2017b
*PHYTOCHROME A*	*PHYA*	C05		A09, C08	Raman et al., 2014; Schiessl et al., 2017b
*PHYTOCHROME B*	*PHYB*	A05	A03, A05, C03, C05		Raman et al., 2013, 2016
*CRYPTOCHROME 2*	*CRY2*		A10	A10	Schiessl et al., 2017b; Raman et al., 2019
*PHYTOCHROME INTERACTING FACTOR 4*	*PIF4*		?		Raman et al., 2019
*GIBBERELLIN 2-OXIDASE 1*	*GA-2-ox-1*	A02			Raman et al., 2013; Jian et al., 2019
*GIBBERELLIN 20-OXIDASE*	*GA-20-ox*		C01		Schiessl et al., 2015
*REPRESSOR OF GA1*	*RGA1*	A02			Li et al., 2018
*SQUAMOSA PROMOTER LIKE 3*	*SPL3*		A03	A05	Raman et al., 2016; Schiessl et al., 2017b

*^?^Location not reported.*

### The Central Hub: FT, FD, and SOC1

**FIGURE 1:**
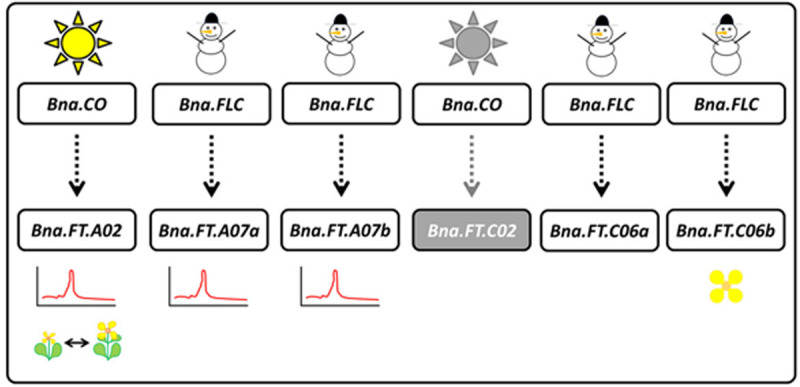
Model of *Bna.FT* regulation based on available literature data (references see main text). *Bna.FT.C02* is most likely to be a pseudogene (gray). *Bna.FT.C06b* mutation was shown to influence flowering time (flower). *Bna.FT* copies on A02 and A07 were found in flowering time QTL (LOD plot). Promoter and gene expression analysis indicates that *Bna.FT.A02* (and unlikely *Bna.Ft.C02*, if expressed) responds to *Bna.CO* and day length regulation (sun), while copies on A07 and C06 respond to *Bna.FLC* and vernalization (snowman). *Bna.FT.A02* was also found to underlie a selective sweep between winter, semi-winter, and spring material (two contrasting rapeseed plants).

The major flowering regulator *FLOWERING LOCUS T (FT)* has six copies in *B. napus*, located on chromosomes A02, A07 (two copies), C02 and C06 (two copies) (Wang J. et al., 2009). BLAST positions in the first published reference genome, however, were partially different and assigned one copy to C04, which could be misassembly (Schiessl et al., 2017b). *Bna.FT.C02* was reported to be pseudogenized, obviously due to a transposon insertion into its promoter region (Wang et al., 2012). Non-expression of *Bna.FT.C02* was later confirmed in a winter type (Guo et al., 2014), while others found flowering time QTL (Rahman et al., 2018) and reported expression in spring type (Raman et al., 2019). The same study found a correlation between the expression of all copies with flowering time, at least in spring type *B. napus* (Raman et al., 2019). Several copies of *Bna.FT* (on A02 or A07) were found in major QTL intervals in different studies, all of them in populations derived from spring type rapeseed (Raman et al., 2013, 2019; Nelson et al., 2014). EMS mutants of *Bna.FT.C06b*, but not of *Bna.FT.C06a* changed flowering time in a winter type (Guo et al., 2014). The copies on A07 and C06 are located within inverted duplicated regions, indicating they arose from a tandem duplication before the speciation separating *B. rapa* and *B. oleracea* (Wang J. et al., 2009). Regulatory regions of *Bna.FT.A02* and *Bna.FT.C02* were lacking a binding site known to be important for binding of the vernalization regulator *FLC*, the so-called CArG box, but were containing a binding motif for the photoperiod regulator *CONSTANS (CO)* (Wang J. et al., 2009; Raman et al., 2019). The copies on A07 and C06 showed the opposite pattern (Wang J. et al., 2009; Raman et al., 2019). This indicates that regulation via vernalization and regulation via photoperiod might have been split between the A02/C02 copies and the A07/C06 copies, although this has not been demonstrated yet. An extensive study on natural variation in almost 1,000 *B. napus* accessions found that the second strongest selection signature between winter and spring type *B. napus* was located in a region harboring *Bna.FT.A02* (Wu et al., 2018). A region 3 kb upstream of this copy also showed the strongest GWAS peak for flowering time in the same study, indicating promoter variation accounts for the effect (Wu et al., 2018). Indeed *Bna.FT.A02* expression was different between winter, semi-winter and spring material (Wu et al., 2018). Another study found that *Bna.FT.A02* expression was not released directly after vernalization, but only later shortly before BBCH60, beginning of flowering (Guo et al., 2014). The A07/C06 copies, however, responded directly to vernalization (Guo et al., 2014). Together with the promoter motif analysis, this indicates that the A07/C06 copies may be majorly regulated by vernalization (*Bna.FLC*), while the A02 copy may be majorly regulated by day length (*Bna.CO*) (see Figure 1 for a model). Interestingly, no functional variation for the coding regions of *Bna.FT.A02* and *Bna.FT.C02* was found across 280 accessions of *B. napus*, indicating expression variation was exclusively due to promoter variation (Schiessl et al., 2017b). The same study also found that *Bna.FT.A02* was never affected by a deletion event, supportive of this copy being essential (Schiessl et al., 2017b). *Bna.FT.A02* was also found to react to drought stress in winter type rapeseed (Schiessl et al., 2020), possibly via the age pathway. A recent study comparing *Bna.FT.A02* expression in early and late winter type rapeseed found that there may be genotypic variance in the responsiveness to vernalization, as the early flowering Cabriolet was able to upregulate *Bna.FT.A02* in response to vernalization, while the late flowering genotype Darmor was not or only slightly (Tudor et al., 2020).

The protein binding partner of *FT, FLOWERING LOCUS D (FD)* (note: there’s also another gene named the same way, but abbreviated as FLD working in the autonomous pathway) has received much less attention than *FT*, and has also not be named as a candidate gene in any of the numerous QTL studies for flowering time up to date. Two copies, *Bna.FD.C01* and *Bna.FD.C03*, showed distinct patterns of allelic variation in swedes, indicating they would contribute to the differential flowering behavior of this subspecies (Schiessl et al., 2017b).

The second most important floral integrator, *SUPPRESSOR OF OVEREXPRESSION OF CONSTANS 1 (SOC1)* is also not well studied in *B. napus*. No data is available on *Bna.SOC1* expression patterns for its six copies. One of its copies, *Bna.SOC1.A05*, was found to be highly conserved across 280 diverse *B. napus* accessions, possibly indicating special functional importance (Schiessl et al., 2017b). Analysis of promoter sequences of different *Brassica SOC1* homologs point into the same direction (Sri et al., 2020). The authors found that all six *B. napus SOC1* promoter sequences show distinct patterns of transcription factor binding sites, similar to the diploid *Brassica* species and *B. juncea* (Sri et al., 2020). In *B. rapa* and *B. juncea*, the resulting expression patterns were markedly different, with differences in expression between tissues (for copies on A05 and A03) and in total expression level and response (for the A04 copy) (Sri et al., 2020). In *B. juncea*, the patterns of transcription factor binding sites were correlating with the respective expression patterns and with expression of putative regulators (Sri et al., 2020), showing elegantly how post-polyploidization diversification in promoter sequences can lead to subfunctionalization.

### Vernalization: FLC, FRI, and VIN3

**FIGURE 2:**
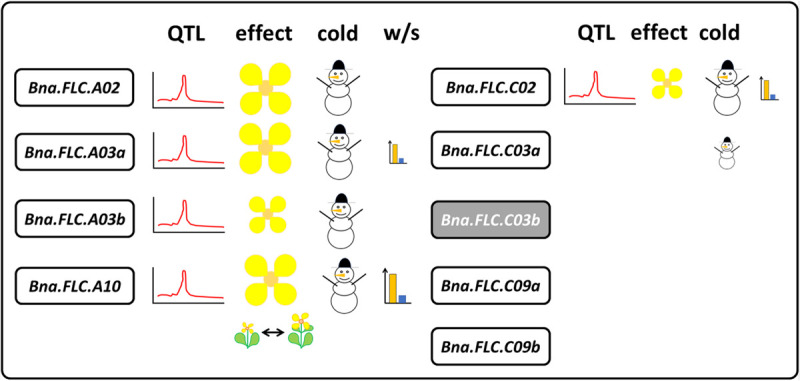
Summary of *Bna.FLC* copy functionality based on literature data (references see main text). *Bna.FLC.C03b* is most likely a pseudogene (gray). Copies on A02, A03, A10, and C02 have been found in QTL for flowering time (LOD plot). The same copies were able to complement the *Arabidopsis* flc mutation (flower, size indicates effect). The same copies were also shown to be downregulated under cold (snowman), while *Bna.FLC.C03a* still showed partial downregulation. *Bna.FLC.A03b*, *Bna.FLC.A10*, and *Bna.FLC.C02* have been shown to be differentially expressed between winter and spring material (Barplot, size indicates degree), but only *Bna.FLC.A10* was located in a selective sweep between winter and spring (gene expression co-segregating) and between swede and non-swede (two contrasting rapeseed plants).

Most studies on *B. napus* flowering time genes have been performed on the main vernalization regulator *Bna.FLC*, mostly because it was recognized as a candidate gene for many flowering time QTL quite early on (Quijada et al., 2006; Udall et al., 2006; Fletcher et al., 2014; Nelson et al., 2014; Raman et al., 2016). *Bna.FLC* has nine annotated copies in the *B. napus* genome, located on chromosomes A02, A03 (two copies), A10, C02, and C03 (two copies), and C09 (two copies) (Zou et al., 2012). Moreover, an incomplete copy on A01 has also been reported, most likely a duplicate of the A02 copy (Schiessl et al., 2019a). Early transformation studies indicated not all of them have the same effect, with the copy on A10 having the strongest effect on vernalization (Tadege et al., 2001). Later on, it was found that this effect was due to a MITE transposon insertion into the promoter of *Bna.FLC* increasing *Bna.FLC* expression in winter type rapeseed (Hou et al., 2012). While semi-winter material originally showed a comparable *Bna.FLC.A10* expression, downregulation in response to cold happened much quicker than in a winter type, presumably because the transposon sequence interfered with the mechanism of downregulation (Hou et al., 2012). In spring type accessions, *Bna.FLC.A10* expression was found to be low even before vernalization (Wu et al., 2018; Schiessl et al., 2019a). Wu et al. (2018) subsequently also found that the strongest selection signal between winter and spring accessions was located close to the A10 copy, linked to differential expression between winter and spring material, a finding confirmed by others (Schiessl et al., 2017b, 2019a). At the same time, *Bna.FLC.C03b* was found to be a pseudogene (Zou et al., 2012; Schiessl et al., 2019a). Meanwhile, the role of the other seven copies remains unclear. Both copies on C09 and partly also *Bna.FLC.C03a* were found to have lost their cold responsiveness (Schiessl et al., 2019a). However, *Bna.FLC.A02*, *Bna.FLC.A03ab*, and *Bna.FLC.C02* as well as *Bna.FLC.A10* are downregulated by cold (Raman et al., 2016; Schiessl et al., 2019a). Raman et al. claim that FLC2 (*Bna.FLC.A02/C02*) is responsible for 22% of the flowering time variation in non-vernalized conditions in a diverse population of spring type rapeseed (Raman et al., 2016). Only *Bna.FLC.A03a* and *Bna.FLC.C02* show some degree of differential expression between winter and spring, although this seems to be dependent on the age of the sampled leaf material (Schiessl et al., 2019a). Varying tissue-specific differences in *Bna.FLC* expression have been reported before (Zou et al., 2012). The data indicate that *Bna.FLC.A10* is not only responsible for the high vernalization dependency of winter types, but also for the moderate vernalization dependency of semi-winter types. A recent study identified additional transposon insertions influencing expression in the promoters of *Bna.FLC.A10* and *Bna.FLC.A02* and concluded that *Bna.FLC.A10* determines growth type in combination with *Bna.FLC.A02* (Yin et al., 2020). Interestingly, swedes (ssp. *napobrassica*), showing an extremely strong vernalization dependency, were reported to carry two copies of *Bna.FLC.A10*, possibly as a result of selection against bolting (Schiessl et al., 2017b). In case of monomorphic *Bna.FLC.A10*, other *Bna.FLC* copies (A02, A03, and C02) may still show up as modulating flowering time factors in non-vernalized conditions. Likewise, *Bna.FLC.A02*, *Bna.FLC.A03b*, and *Bna.FLC.C02* seem to be influential on flowering time in spring material, although they are only able to delay, but not to inhibit flowering. This was recently confirmed for *Bna.FLC.A02* (Tudor et al., 2020) (Figure 2).

In *A. thaliana*, vernalization requirement was attributed to variation in either *FLC* or *FRIGIDA (FRI*) (Jiang et al., 2009; Choi et al., 2011). *FRI* has four copies in the *B. napus* genome, of which one of them, *Bna.FRI.A03*, was associated to flowering variation quite early on (Wang et al., 2011). The same copy was later on found to be associated to the subspecies differentiation of swedes, but also for the winter-spring split (Schiessl et al., 2017b). All four copies are expressed (Wang et al., 2011; Schiessl et al., 2019a), and the expression level was found to be comparable between copies, growth types and seemed to be independent of vernalization, but the copies named *BnaA.FRI.a* (A03) and *BnaX.FRI.d* showed significant differences in tissue patterning, being expressed mostly in flowers, while showing very low expression in leaves (Wang et al., 2011). The copies on A03 and C03 were slightly upregulated upon cold, but returned to normal levels afterwards (Schiessl et al., 2019a). Four haplotypes which were associated to differential flowering behavior were detected, but not in spring types, loosely in semi-winter and strongly in winter type accessions (Wang et al., 2011). Those haplotypes contained several non-synonymous SNPs and InDels (Wang et al., 2011). A GWAS for flowering time in winter material in 12 different environments detected a respective peak in a Chinese environment with mild winters, indicating it might influence flowering under non-saturated vernalization conditions (Schiessl et al., 2015). A QTL study in spring material detected a QTL in the vicinity of *Bna.FRI.A03*, but no allelic effect of the copy itself (Raman et al., 2013) as well as a GWAS performed predominantly in semi-winter accessions (Raman et al., 2016). So while there is a clear consent that only *Bna.FRI.A03* is influential for flowering time, the reason for this is unclear. As two of the other copies were predicted to have altered protein structures as compared to *A. thaliana* (Wang et al., 2011), these copies might already have attained a different role.

Another vernalization gene which has been brought up as a candidate gene to influence flowering time in oilseed rape is *VERNALIZATION INSENSITIVE 3 (VIN3)* (Shi et al., 2009; Nelson et al., 2014; Schiessl et al., 2017b; Shah et al., 2018). It has four copies in *B. napus*, of which mostly the A02 copy was attributed to phenotypic effects (Shi et al., 2009; Nelson et al., 2014; Schiessl et al., 2017b; Shah et al., 2018). However, gene expression analysis in a spring accession (Westar) over time showed that all four copies get upregulated during cold in both leaf and apex, as expected from *A. thaliana*, and get downregulated upon return to warmer temperatures (Schiessl et al., 2019a). *Bna.VIN3.C03* had the lowest expression, while the others showed comparable expression levels. This would indicate a low level of subfunctionalization in terms of gene expression, and the reason why only *Bna.VIN3.A02* has been found as candidate might be attributed to existing variation in coding regions. Analysis of SNP distribution in *B. napus* populations of winter, spring and swede growth types found *Bna.VIN3.A02* as candidate for the winter-spring split, and *Bna.VIN3.A03* as a candidate for the swede split (Schiessl et al., 2017b). However, no non-synonymous SNP was found to associate with this pattern (Schiessl et al., 2017b), contradicting this hypothesis. Works in *B. oleracea* (cauliflower) have shown that the dynamics of upregulation in *Bol.VIN3* instead of absolute expression level are decisive for flowering time variation (Ridge et al., 2015). More precise time series of *Bna.VIN3* expression in cold are therefore needed to judge the degree of subfunctionalization between the *Bna.VIN3* copies.

### Autonomous Pathway

The autonomous pathway in *A. thaliana* is regulating *FLC* mRNA concentration independently of cold (Cheng et al., 2017). The autonomous pathway gene *Bna.FY* was named particularly often in QTL studies before the publication of the *B. napus* reference genome, as many candidate regions showed a BLAST hit to “*a region at the top of chromosome 5 containing the flowering time genes FLC, FY*, *and CO*” (Raman et al., 2013; Fletcher et al., 2014; Luo et al., 2014); however, in most cases the major effects might rather have been due to *FLC* or *CO* than to *FY*. However, Jian et al. (2019) found seven members of the autonomous pathway were differentially expressed between parents of their RIL population and at the same time located in a respective flowering time QTL, also including *Bna.FY*, indicating it might still contribute to flowering time variation. Some other homologs of autonomous pathway genes have also been named as candidate genes for flowering time in *B. napus*, like *FLD* (Raman et al., 2016; Jian et al., 2019), *AGL18* (Raman et al., 2016), or two copies of *LD* (Schiessl et al., 2015). However, data allowing a deeper insight into copy-specific regulation or involvement is not available up to now, and much is left to reveal for autonomous pathway genes in *B. napus*.

### Photoperiod: CO, GI, SRR1, PHY, and CRY

In contrast to vernalization, the photoperiod behavior of rapeseed is not well studied. It is known that rapeseed does generally not flower at a day length of 8 h (most vernalization chambers run at this day length). However, in spring rapeseed, 24% of accessions were able to flower at 8 h day length (Raman et al., 2019), while in a different study, spring rapeseed was generally reported to flower at a day length of 10 h, although strongly delayed (Rahman et al., 2018). In the same study, flowering time was not different between 14 h day length and 18 h day length, so the critical day length at least for spring rapeseed lies between 10 and 14 h of light (Rahman et al., 2018). Others found that there is genotypic variation for critical day length between accessions, ranging between 10 and 12 h (Luo et al., 2018). However, in field trials, photoperiod is often confounded with temperature, so the exact influence of photoperiod remains elusive to date.

At the same time, not a lot is known about the main photoperiod pathway gene *Bna.CO*, although it was one of the first *B. napus* flowering time genes to be investigated (Robert et al., 1998). This early study identified four copies which were all expressed (Robert et al., 1998). One of them was able to complement the *co-1* mutation in *A. thaliana* (Robert et al., 1998). When a reference genome for *B. napus* became available, six copies of *Bna.CO* were identified, along with four copies of *CO-like* genes, which complicate the analysis (Schiessl et al., 2014). Copies of *Bna.CO* or *Bna.CO-like* were named as candidate genes for flowering time QTL on A02 (Nelson et al., 2014) A10 (Quijada et al., 2006), C01 (Rahman et al., 2018), C03 (Li et al., 2018) and C09 (Xu et al., 2016). Copies of *Bna.CO* and *Bna.CO-like* on C09 with respective non-synonymous SNP variation were also found in regions separating swedes from non-swede material (Schiessl et al., 2017b). Although not every candidate gene might turn out as a true reason for phenotypic variance, the diversity of gene loci detected does not point to substantial subfunctionalization. This would in turn mean that gene dosage and therefore the number of loci able to produce functional protein could play a larger role here. *Bna.CO* copies on the C genome were found to be stable in copy number, while the other copies showed considerable variation (Schiessl et al., 2017b). When comparing gene expression between early and late flowering semi-winter accessions, no difference for any *Bna.CO* copy was found (Jian et al., 2019), however, the samples were taken at 10 am, where CO expression is normally still low. To our knowledge, more data on gene expression, subfunctionalization or protein stability have not been raised, and further conclusions on this important flowering time regulator are not possible to date.

In *A. thaliana*, *CO* transcription is repressed by CDF proteins, which in turn are negatively regulated by GIGANTEA (GI) and FKF1 (Johansson and Staiger, 2015). *Bna.CDF1* and *Bna.CDF2* were found to be down-regulated in early flowering semi-winter rapeseed relative to late flowering semi-winter in the morning, although single copies showed the opposite behavior (Jian et al., 2019). *Bna.CDF1* has four copies in the *B. napus* genome, of which two were found to vary strongly in copy number (Schiessl et al., 2017b).

*CDF1* seems to be controlled by a novel protein called SENSITIVITY TO RED LIGHT REDUCED 1 (SRR1) (Johansson and Staiger, 2014). In *B. napus*, *Bna.SRR1* has five copies, of which two (on A03 and A10) were not expressed (Schiessl et al., 2019b). The remaining three showed differential expression patterns between spring and winter type rapeseed (Schiessl et al., 2019b), in line with the finding that *Bna.SRR1.A02* was found to be a candidate for the winter-spring split earlier (Schiessl et al., 2017b). *Bna.SRR1.A02* and *Bna.SRR1.C02* were also able to complement the respective knockout mutant in *A. thaliana*, while *Bna.SRR1.C09* was not (Schiessl et al., 2019b). *Bna.SRR1.C09* carried a deletion of several amino acids in a putatively important region (Schiessl et al., 2019b). It is therefore highly likely that the gene copies underwent subfunctionalization, with *Bna.SRR1.A02* being the most functionally conserved copy, influencing flowering time via *CDF1* (Schiessl et al., 2019b).

**FIGURE 3:**
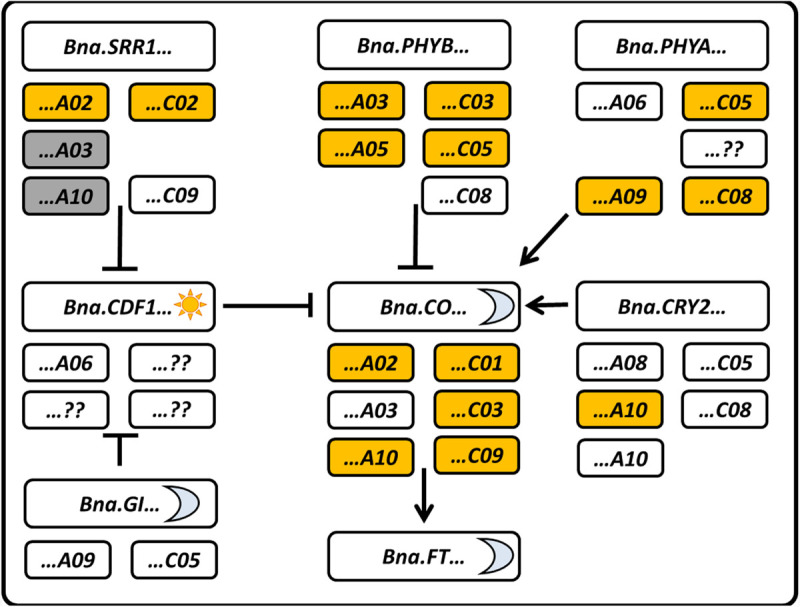
Schematic representation of selected genes from the photoperiodic pathway with their locations in *B. napus*. Gray boxes indicate pseudogenes, yellow boxes indicate this copy has been named as a candidate in a GWAS, QTL, or selective sweep analysis. Thy sun symbol marks a gene which peaks in the morning, while the moon indicates it peaks in the evening as inferred from *A. thaliana*. Arrows and blunt end indicate activation and inhibition, respectively.

In *Arabidopsis*, CO is further regulated post-translationally via the action of several ubiquitin-E3-ligases and photoreceptors like phytochromes and cryptochromes. Interestingly, *Bna.PHYA*, *Bna.PHYB*, and *Bna.CRY2* all retained the same copy number (five copies) in *B. napus* (Schiessl et al., 2017a). Both *Bna.PHYB*, a negative regulator of CO protein stability (Raman et al., 2013, 2016) as well as the positive regulators *Bna.PHYA* (Raman et al., 2014) and *Bna.CRY2* (Raman et al., 2019) were found to be candidates for flowering time in different QTL and GWAS studies. A structural rearrangement encompassing the A09 and C08 copies of *Bna.PHYA* was found to be associated to the swede morphotype, along with allelic variance in *Bna.CRY2.A10* (Schiessl et al., 2017b), likely to represent adaptation of swede flowering to longer days. Data on transcription or protein levels of those genes in *B. napus* are not available, so no conclusion on subfunctionalization or mode of action can be drawn so far (Figure 3).

### Ambient Temperature: PIF4

In *Arabidopsis*, *FT* expression is further gated by binding of *PHYTOCHROME INTERACTING FACTOR 4 (PIF4)* to the *FT* promoter, which can only bind in warmer temperatures when the chromatin carries less H2A.Z histone (Wigge, 2013). *PIF4* itself is also under transcriptional control of the circadian clock (Wigge, 2013). A copy of *Bna.PIF4* was recently implicated in a photoperiod-sensitive flowering time QTL (Raman et al., 2019). In *B. rapa*, however, it was found that *Bra.FT.A02* expression was decreased at warmer temperatures (28°C) compared to normal (21°C), obviously via an increase in H2A.Z in *Bra.FT.A02* chromatin, resulting in later flowering (Del Olmo et al., 2019). Spring rapeseed growing at 18°C/8°C day/night cycles still flowered slightly later than plants at constant 20°C at the same photoperiod (16 h) (Rahman et al., 2018), so there seems to be an optimum temperature above 20°C and well below 28°C. Moreover, this indicates that temperature regulation in *Brassica* is different from what we observe in *A. thaliana*, and respective studies need to be carried out to dissect this trait in *B. napus*.

### Gibberellins, Age, and Stress: SPL and DELLA

Two pathways regulate flowering time in absence of inductive conditions: the gibberellin (GA) pathway and the miR156/SPL module (Yu et al., 2012). GAs are negative regulators of DELLA proteins, which are interacting with SPLs, so there is interaction between both pathways (Yu et al., 2012). The miR156/SPL module is known as the age pathway, which is mediated via several highly conserved micro RNAs (miRNAs) like miR156 and miR172 (Wang J.-W. et al., 2009; Wang, 2014). miR156 is a negative post-transcriptional regulator of many different *SQUAMOSA PROMOTER LIKE (SPL)* genes (Wang J.-W. et al., 2009). In seedlings, miR156 levels are high, but steadily decrease with increasing plant age, and SPL repression is more and more released (Wang J.-W. et al., 2009; Wang, 2014). SPL9 induces expression of miR172, which in turn shuts off negative flowering regulators, while SPL3 directly activates downstream flowering time genes like *LFY* (Wang J.-W. et al., 2009; Yamaguchi et al., 2009).

In *B. napus*, two different GA synthesis enzymes have been found in QTL regions for flowering time: *GA-2-ox-1* (Raman et al., 2013; Jian et al., 2019) and *GA-20-ox* (Schiessl et al., 2015), along with the DELLA protein RGA1 (Li et al., 2018; Jian et al., 2019). While miRNAs were not considered or reported in any QTL region in *B. napus*, majorly due to the lack of suitable miRNA gene annotation, one study reports *Bna.SPL3.A03* to be located in a QTL region in spring material (Raman et al., 2016), while another claims the same copy to be a pseudogene (Schiessl et al., 2017b). Copies of DELLA proteins (A09, C09), *Bna.GA-3-ox.A06* and *Bna.SPL3.A05* were also found in selective sweeps between swede and non-swede material (Schiessl et al., 2017b). *Bna.SPL* has six copies in *B. napus*, of which one is a pseudogene, while all others carry some type of variation (Schiessl et al., 2017b). The relative lack of respective gene copies in QTLs might reflect the fact that most flowering time QTL studies take place under inductive conditions.

Interestingly, however, most flowering time genes affected under drought stress in *B. napus* were found to belong to the gibberellin and age pathway, which indicates that stress signaling might take this route to regulate flowering in response to abiotic stress (Schiessl et al., 2020). Candidates to mediate this response are, among others, miR156s, which were found to be differentially expressed under drought stress, possibly in reaction to altered sugar level under photosynthetic limitations (Schiessl et al., 2020). However, data from this study also confirmed the high complexity of those interactions in *B. napus*, as different copies of the same gene seemed to react differentially to the same miRNA level (Schiessl et al., 2020). This points to regulatory co-evolution of specific miRNA-gene pairs and stresses the strong need to perform studies on gene copy level in *B. napus* – inferring gene function from *A. thaliana* is a good start, but does not provide enough information on breeding targets.

## Discussion

Although much progress has been achieved to shed light on flowering time regulation in rapeseed, we still lack answers to important questions in regards to the specific situation in this polyploid oil crop. Major genetic effects on traits like vernalization dependency have been characterized down to gene copy level and revealed considerable subfunctionalization. Other pathways, however, like photoperiod and temperature signaling, still remain largely obscure in this respect. Moreover, the few studies existing worked mainly with spring type rapeseed, and data on the same traits in semi-winter and winter rapeseed are scarce. More and more targeted studies will be necessary to provide reliable data for breeding programs, under consideration of cross-pathway effects, the influence of the circadian clock, the genetic background and epigenetic regulation. In the light of shifting climate zones, influences by day length, ambient temperature, and drought stress need more attention. While the close relationship to the model was and is very helpful for hypothesis development, the current review shows clearly that knowledge cannot be directly inferred from the model to the crop, making functional genetic studies in crops unreplaceable. On top of providing invaluable information for breeding programs, such data will also improve our understanding of post-polyploidization adaptation as an evolutionary principle.

## Author Contributions

SS conceptualized and wrote the manuscript.

## Conflict of Interest

The authors declare that the research was conducted in the absence of any commercial or financial relationships that could be construed as a potential conflict of interest.
